# Induction of Neuronal Differentiation of Murine N2a Cells by Two Polyphenols Present in the Mediterranean Diet Mimicking Neurotrophins Activities: Resveratrol and Apigenin

**DOI:** 10.3390/diseases6030067

**Published:** 2018-07-22

**Authors:** Amira Namsi, Thomas Nury, Haithem Hamdouni, Aline Yammine, Anne Vejux, Dominique Vervandier-Fasseur, Norbert Latruffe, Olfa Masmoudi-Kouki, Gérard Lizard

**Affiliations:** 1 Team Bio-PeroxIL, ‘Biochemistry of the Peroxisome, Inflammation and Lipid Metabolism’ (EA7270)/University Bourgogne Franche-Comté/Inserm, 21000 Dijon, France; amira.namsi@gmail.com (A.N.); thomas.nury@u-bourgogne.fr (T.N.); haythem.hamdouni@gmail.com (H.H.); alineyammine5@gmail.com (A.Y.); anne.vejux@u-bourgogne.fr (A.V.); norbert.latruffe@u-bourgogne.fr (N.L.); 2 UR/11ES09, Lab. ‘Functional Neurophysiology and Pathology’, Faculty of Sciences of Tunis, University Tunis El Manar, Tunis 2092, Tunisia; olfa.masmoudi@fst.utm.tn; 3 LR12SP11 ‘Molecular Biology Applied to Cardiovascular Diseases, Hereditary Nephropathies and Pharmacogenomics’, Dept Biochemistry, CHU Sahloul, Sousse 4000, Tunisia; 4 Bioactive Molecules Research Laboratory, Doctoral School of Sciences and Technologies, Faculty of Sciences, Lebanese University, Beirut 1103, Lebanon; 5 Institut of Molecular Chemistry (ICMUB UMR 6302), Univ. Bourgogne Franche-Comté, 21000 Dijon, France; dominique.vervandier-fasseur@u-bourgogne.fr

**Keywords:** N2a murine neuronal cells, neuronal differentiation, neurotrophic effects, polyphenols, apigenin, resveratrol

## Abstract

In the prevention of neurodegeneration associated with aging and neurodegenerative diseases (Alzheimer’s disease, Parkinson’s disease), neuronal differentiation is of interest. In this context, neurotrophic factors are a family of peptides capable of promoting the growth, survival, and/or differentiation of both developing and immature neurons. In contrast to these peptidyl compounds, polyphenols are not degraded in the intestinal tract and are able to cross the blood–brain barrier. Consequently, they could potentially be used as therapeutic agents in neurodegenerative pathologies associated with neuronal loss, thus requiring the stimulation of neurogenesis. We therefore studied the ability to induce neuronal differentiation of two major polyphenols present in the Mediterranean diet: resveratrol (RSV), a major compound found in grapes and red wine, and apigenin (API), present in parsley, rosemary, olive oil, and honey. The effects of these compounds (RSV and API: 6.25–50 µM) were studied on murine neuro-2a (N2a) cells after 48 h of treatment without or with 10% fetal bovine serum (FBS). Retinoic acid (RA: 6.25–50 µM) was used as positive control. Neuronal differentiation was morphologically evaluated through the presence of dendrites and axons. Cell growth was determined by cell counting and cell viability by staining with fluorescein diacetate (FDA). Neuronal differentiation was more efficient in the absence of serum than with 10% FBS or 10% delipidized FBS. At concentrations inducing neuronal differentiation, no or slight cytotoxicity was observed with RSV and API, whereas RA was cytotoxic. Without FBS, RSV and API, as well as RA, trigger the neuronal differentiation of N2a cells via signaling pathways simultaneously involving protein kinase A (PKA)/phospholipase C (PLC)/protein kinase C (PKC) and MEK/ERK. With 10% FBS, RSV and RA induce neuronal differentiation via PLC/PKC and PKA/PLC/PKC, respectively. With 10% FBS, PKA and PLC/PKC as well as MEK/ERK signaling pathways were not activated in API-induced neuronal differentiation. In addition, the differentiating effects of RSV and API were not inhibited by cyclo[DLeu^5^] OP, an antagonist of octadecaneuropeptide (ODN) which is a neurotrophic factor. Moreover, RSV and API do not stimulate the expression of the diazepam-binding inhibitor (DBI), the precursor of ODN. Thus, RSV and API are able to induce neuronal differentiation, ODN and its receptor are not involved in this process, and the activation of the (PLC/PKC) signaling pathway is required, except with apigenin in the presence of 10% FBS. These data show that RSV and API are able to induce neuronal differentiation and therefore mimic neurotrophin activity. Thus, RSV and API could be of interest in regenerative medicine to favor neurogenesis.

## 1. Introduction

Polyphenols belong to one of the most abundant phytochemicals in the plant kingdom. They are the result of the secondary metabolism of plants through two fundamental metabolic pathways: the shikimate pathway and the acetate pathway [1,2]. There are currently about 8000 different polyphenols, divided into at least 10 different classes based on their chemical structure. They are classified as: (1) flavonoids including flavanols, isoflavones, flavanones, flavonones, and anthocyanidins; and (2) nonflavonoids such as phenolic acids (groups of compounds derived from benzoic and hydroxycinnamic acids), stilbenes, and lignans; tannins are flavonoid polymers [3,4].

Polyphenols, which have nutritional interest as micronutrients, are particularly abundant in several foods (vegetables, fruits), oils (argan and olive oils) and beverages (red wines) associated with in the Mediterranean diet. There is much evidence from in vitro studies, animal models, and clinical studies supporting that polyphenol compounds may have geroprotective activities as well as cytoprotective effects, especially in age-related diseases (cardiovascular diseases, eye diseases (cataracts, age-related macular degeneration) and chronic diseases (bowel diseases)) associated or not with enhanced oxysterol levels [5,6], through the control of mitochondrial dysfunctions, oxidative stress, inflammation, angiogenesis, and cell death [7,8]. At the brain level, phytosterols could prevent oxytosis, i.e., oxidative stress-induced cell death, which could play major role in neurodegeneration [9,10]. There is also lot of evidence that several polyphenols have anti-tumor properties (cell cycle delay, apoptosis induction, metastasis prevention) [11,12]. Interestingly, there is now recent evidence that polyphenols (especially resveratrol, a polyphenol of the stilbene family, found in grapes, blackberries, or peanuts for example) have pro-differentiating properties on different cell types: adipocytes, hematopoietic cells, human umbilical cord mesenchymal stem cells, cancer cells (thyroid, glioblastoma, colon), human lung fibroblasts, keratinocytes, embryonic cardiomyoblasts, and myoblasts [13,14,15,16,17,18]. There is also now evidence that many dietary components of the Mediterranean diet (curcumin, resveratrol, and polyunsaturated fatty acids (PUFAs)), and diets enriched with polyphenols and PUFAs, as well as caloric restriction, physical exercise, and learning, are able to induce neurogenesis in the adult brain. It is therefore tempting to speculate that nutritional approaches, functional foods enriched in polyphenols, or functionalized polyphenols (polyphenols coupled with nanoparticles) [19,20] could provide promising prospects to stimulate adult neurogenesis and combat neurodegenerative diseases and cognitive decline [21,22].

In addition, several polyphenols, including flavonoids such as baicalein, daidzein, luteolin, and nobiletin as well as non-flavonoid polyphenols such as auraptene, carnosic acid, curcuminoids, and hydroxycinnamic acid derivatives including caffeic acid phentyl ester have neurotrophic effects: they enhance neuronal survival and promote neurite outgrowth in vitro, a hallmark of neuronal differentiation [23]. Flavonoids are also able to induce neuronal differentiation of mouse embryonic stem cells and human pluripotent stem cells [24]. Altogether, these data support the neurotrophic effects of polyphenols. They also support the ability of these molecules to mimic the functions and/or to stimulate the production of neurotrophic factors, which are a family of biomolecules (peptides or small proteins such as brain-derived neurotrophic factor (BDNF), nerve growth factor (NGF), and octadecaneuropeptide (ODN)) capable of favoring the growth, survival, and/or differentiation of both developing and mature neurons [23,25,26]. This is in contrast to peptidyl compounds such as neurotrophins; since polyphenols are not degraded in the intestinal tract and are able to cross the blood–brain barrier [26,27], they could potentially be used as therapeutic agents in neurodegenerative pathologies associated with neuronal loss such as ischemic stroke, Alzheimer’s and Parkinson’s diseases, which require the stimulation of neurogenesis [23,28,29]. At the time, while it is considered that polyphenols can mimic neuroprotective activities, little is still known about the ability of these molecules to favor neuronal differentiation and the associated metabolic pathways. Resveratrol, which is an important component of the Mediterranean diet, has been reported to have antioxidant and antitumor properties, but its effects as a neural plasticity inducer are still debated [30]. Apigenin (a chemical compound of the family of flavones, a subclass of flavonoids) is a major polyphenol of parsley, which is also much consumed in the Mediterranean diet, mainly in the Middle East where it is also used in traditional and folklore medicines [31]. Apigenin is found in thyme, rosemary, celery and chamomile; it is also present in honey [32] as well as in olive oil [33]. At the moment, apigenin has been shown to modulate GABAergic and glutamatergic transmission in cultured cortical neurons [34]. Neuroprotective, anti-amyloidogenic, and neurotrophic effects of apigenin have been reported in an Alzheimer’s disease mouse model (APP/PS1) [17]. These effects were associated with an activation of cyclic adenosine monophosphate response element-binding protein (CREB), characterized by an increased level of phosphorylated CREB [17].

In the present study, based on the ability of polyphenols to mimic the action of neurotrophic compounds (cytoprotection and/or differentiation), we asked whether two major polyphenols present in the Mediterranean diet (trans-resveratrol (RSV) and apigenin (API)) were able to induce neuronal differentiation characterized by neurite outgrowth (dendrite and axon formation) on murine neuroblastoma N2a cells, which are cholinergic cells capable of differentiating into either cholinergic or dopaminergic cells depending on the culture conditions [35,36]. Interestingly, N2a cells express the PAC1 receptor, which is a member of the G-protein coupled receptor (GPCR) superfamily including the metabotropic receptors which bind octadecaneuropeptide (ODN) [37,38]. PAC1 and members of GPCR family activate adenylyl cyclase/cAMP/PKA (via Gs-protein coupling) and phospholipase C (PLC)/DAG/protein kinase C (PKC) (via Gq-protein coupling)-dependent signaling pathways [39,40]. The PAC1 receptor also triggers the activation of several other protein kinase cascades such as ERK1/2, JNK1/2, p38 MAPK and PKB [41,42,43]. Consequently, N2a cells have the ability to bind the pituitary adenylate cyclase-activating polypeptide (PACAP), which is widely distributed in the brain and peripheral organs and displays high affinity for the PAC1 receptor [40,44]. They can also be used to study other neuropeptides or molecules (natural or synthetic) capable of interacting with receptors of the GPCR superfamily. Thus, N2a cells constitute a suitable model to study neuronal differentiation and to identify the signaling pathways associated with this process. In this study, the effects of RSV and API on the neuronal differentiation of N2a were compared with those of trans-retinoic acid (RA) used as positive control. To this end, N2a cells were cultured without or with 10% FBS (conventional culture condition) since it is known that various factors present in FBS can modulate cell differentiation. Interestingly, RSV and API trigger the neuronal differentiation of N2a cells.

## 2. Materials and Methods

### 2.1. Cell Culture and Treatments

Mouse neuro-2a (N2a) neuroblastoma cells (ATCC^®^ CCL-131™) were purchased from the American Type Culture Collection (Rockville, MD, USA). N2a cells were plated at a density of 34 × 10^3^ cells/cm^2^; they were cultured in Dulbecco’s modified Eagle medium (DMEM) with high glucose (4.5 g/L), stable glutamine, and sodium pyruvate (Dominique Dutscher, Brumath, France) supplemented with 10% (*v*/*v*) fetal bovine serum (FBS, Pan Biotech, Aidenbach, Germany) and containing 1% (*v*/*v*) antibiotics (100 U/mL penicillin, 100 mg/mL streptomycin) (Pan Biotech). N2a cells were incubated at 37 °C in a 5% CO_2_ humidified atmosphere and passaged twice a week. N2a cells are broadly used to study the neuronal differentiation mechanism and neurite outgrowth [45].

To evaluate the cytotoxicity and the differentiating properties of resveratrol (RSV), apigenin (API), and retinoic acid (RA), 12 × 10^4^ N2a cells were cultured per well in 6-well plates (FALCON, Becton Dickinson, NJ, USA) or in tissue culture dishes (35 × 10 mm, FALCON) containing 1 mL of culture medium with 10% FBS. After 24 h of cell culture, the culture medium was removed, and the cells were cultured for 48 h in the absence or presence of polyphenol (RSV, API) or RA used in a range of concentrations from 6.25 to 50 µM in culture medium without or with 10% FBS. RSV (corresponding to trans-resveratrol), API, and RA (corresponding to trans-retinoic acid) were from Sigma-Aldrich (St Quentin-Fallavier, France). Absolute ethanol (EtOH; Carlo Erba Reagents, Val de Reuil, France) was used as vehicle to dissolve RSV, whereas dimethyl sulfoxide (DMSO; Sigma-Aldrich) was used as vehicle to dissolve API and RA. RA was used as positive control to induce neuronal differentiation as it is well established that all-trans RA induce N2a differentiation into neuronal cells characterized by neurite outgrowth [46].

When the cells were simultaneously treated with the inhibitors H89 (20 µM), U73122 (1 µM), chelerythrine (1 µM) (Sigma-Aldrich), and U0126 (20 µM) (Calbiochem, San Diego, CA, USA), which contain PKA, PLC, PKC and MEK inhibitors, respectively, these compounds were introduced in the culture medium 30 min before RSV, API, or RA. H89 was prepared as a stock solution at 1 mM in distilled water; U0126, U73122 and chelerythrine were prepared as a stock solution in DMSO at 0.1 mM, 0.1 mM, and 1 mM, respectively. To evaluate the involvement of the metabotropic receptor in N2a cell differentiation, the metabotropic receptor antagonist cyclo_1–8_[DLeu^5^] OP (10^−6^M) was added in the culture medium 30 min before RSV, API, or RA.

### 2.2. Evaluation of Neuronal Differentiation with Morphological Criteria

Morphological criteria were used to evaluate neuronal differentiation [47,48,49] on N2a cells cultured in 6-well plates. These criteria were defined on N2a cells with the use of ODN, which is known to trigger neuronal cytoprotection and is capable of inducing differentiation on N2a cells. It is therefore considered as a neuroprotectin [50]. After 24 h of culture in DMEM with 10% FBS, the culture medium was removed and N2a cells were cultured for 48 h in the presence of very low concentrations of ODN (10^−14^ and 10^−12^ M) without FBS (0% FBS) or with 10% FBS. N2a cells (morphologically evocating neuroblasts) have the ability to differentiate in young immature and mature neurons. Under treatment with ODN, differentiated N2a cells with dendrites (D), axons (A) and dendrites + axons (D + A) were observed (Supplementary Figure S1).

These morphological criteria were further used to evaluate the ability of RSV and API to induce neuronal differentiation comparatively to RA used as a positive control. Morphological changes were evaluated after 48 h of culture, in DMEM without FBS (0% FBS), in the absence or presence of RSV (12.5 µM), API (12.5 µM), and RA (6.25 µM), and in DMEM with 10% FBS in the absence or presence of RSV (12.5 µM), API (12.5 µM) and RA (25 µM). Cells were observed by phase contrast microscopy under an inverted Zeiss microscope (Primovert, Jena, Germany) at a 20× magnification (Objective: LD Plan-Achromat, ref: 415500-1614-000). Digitalized images were obtained with a Zeiss camera (5MP HD IP). Neuronal differentiation was determined on 20 images corresponding to 20 microscopical fields (5 × 4) taken at the center of the culture dish. N2a cell differentiation was quantified by neurite outgrowth (dendrites and/or axons). The percentages of differentiated cells with dendrites, axons, and dendrites + axons were determined. All assays were performed at least in four independent experiments.

#### 2.2.1. Cell Counting

N2a cells were cultured in 6-well plates. N2a cells previously cultured for 24 h in DMEM were further cultured for 48 h in DMEM without or with 10% FBS in the absence or presence of RSV, API, and RA (6.25 to 50 µM). At the end of the treatment, adherent cells were collected by trypsinization and the total number of cells was determined by using a hemocytometer. All assays were realized at least in four independent experiments.

#### 2.2.2. Measurement of Cell Viability with the Fluorescein Diacetate (FDA) Test

N2a cells, previously cultured for 24 h in DMEM with 10% FBS in 6-well plates, were further incubated for 48 h without or with 10% FBS in the absence or presence of RSV, API, and RA (6.25 to 50 µM). At the end of the treatment, cells were incubated for 10 min at 37 °C with 15 µg/mL fluorescein diacetate (FDA, Sigma-Aldrich), rinsed twice with phosphate buffer saline (PBS) and lysed with a Tris/HCl solution containing 1% sodium dodecyl sulfate (SDS) (Sigma-Aldrich). The FDA test is a cell viability test. Fluorescence was measured with excitation at 485 nm and emission at 528 nm using a plate reader (Tecan Sunrise, Tecan, Lyon, France). All assays were performed at least in four independent experiments.

#### 2.2.3. Flow Cytometric Analysis of Cell Cycle

Flow cytometric analyses were carried out after staining with propidium iodide (PI) to determine the impact of RSV, API, and RA on the repartition of the cells in the different phases of the cell cycle. To this end, N2a cells previously cultured for 24 h in DMEM with 10% FBS were further cultured for 48 h either in DMEM without FBS, in the absence or presence of RSV (12.5 µM), API (12.5 µM), and RA (6.25 µM), or in DMEM with 10% FBS in the absence or presence of RSV (12.5 µM), API (12.5 µM), and RA (25 µM). At the end of the treatment, cell cycle was realized on adherent cells collected by trypsinization. Cells were washed with PBS, and 1–2 × 10^6^ cells were stained with PI as previously described [51]. Cells were resuspended in 80% cold ethanol (2 h, −20 °C), washed in PBS, and resuspended in 300 µL of PBS containing 80 µg/mL PI and 200 µg/mL RNase A. After 1 h of incubation (37 °C, 1 h), 1–2 mL of PBS were added, and flow cytometric analyses were performed on a Galaxy flow cytometer (Partec, Münster, Germany). Fluorescence of PI was collected using a 590/10 nm bandpass filter and measured on a linear scale. Subsequently, 10,000 cells were acquired, and data were analyzed with Flomax software (Partec).

#### 2.2.4. Real-Time PCR Analysis

Total RNAs from N2a cells obtained after 48 h of treatment with RSV (12.5 µM), API (12.5 µM) and RA (6.25 and 25 µM) without or with 10% FBS, were extracted and purified with the RNeasy Mini Kit (Qiagen, Courtaboeuf, France). The concentration of RNA was measured by spectrophotometry (UV-1800, Shimadzu, Kyoto, Japan) at an absorbance of 260 nm and calculated with UV Probe (Shimadzu software). Then, 1 µg of total RNA from each sample was converted into single-stranded cDNA using the iScript cDNA kit (BioRad, Marne la Coquette, France) according to the protocol reaction provided by the manufacturer: 5 min at 25 °C, 20 min at 46 °C, and 5 min at 95 °C. cDNA was then amplified in the presence of FG Power SYBR Green (Thermo Fischer Scientific, Illkirch-Graffenstaden, France) and 100 µM of forward and reverse mouse primers (Eurogentec, Liége, Belgium):

* Diazepam-binding inhibitor (DBI) sequences: forward (*5′-gaagcgcctcaagactcagc-3′*) and reverse (*5′-ttcagcttgttccacgagtcc*-*3′*),

* Nerve growth factor (NGF) sequences: forward (*5′*-*acactctgatcactgcgtttttg*-*3′*) and reverse (*5′*-*ccttctgggacattgctatctgt*-*3′*),

* Brain-derived neurotrophic factor (BDNF) sequences: forward (*5′*-*atggttatttcatacttcggttgca*-*3′*) and reverse (*5′*-*agctgggtaggccaagttg*-*3′*),

* 36B4 sequences (*36B4* was used as reference gene): forward (*5′*-*gcgacctggaagtccaacta-3′*) and reverse (*5′*-*atctgcttggagcccacat-3′*)

StepOne Plus (Life Technologies/Thermo Fischer Scientific, Courtaboeuf, France) was used to detect the real-time quantitative PCR products of reverse-transcribed cDNA samples according to the manufacturer’s instructions. The incubation conditions were as follows: 90 °C for 20 s, followed by 40 cycles (95 °C for 30 s, 60 °C annealing for 30 s) and a cycle from 60 °C to 95 °C. Specific amplification efficiencies were calculated with StepOne software. Results were expressed as means of Ct (threshold cycle value) ± standard deviation (SD).

#### 2.2.5. Statistical Analysis

Statistical analysis was performed using the GraphPad Prism5 software (San Diego, CA, USA). An ANOVA test followed by a Mann–Whitney test was used. A *p* value of 0.05 or less were considered as statistically significant.

## 3. Results

### 3.1. Evaluation and Quantification of Resveratrol- and Apigenin-Induced Neuronal Differentiation of N2a Cells

**Figure 1:**
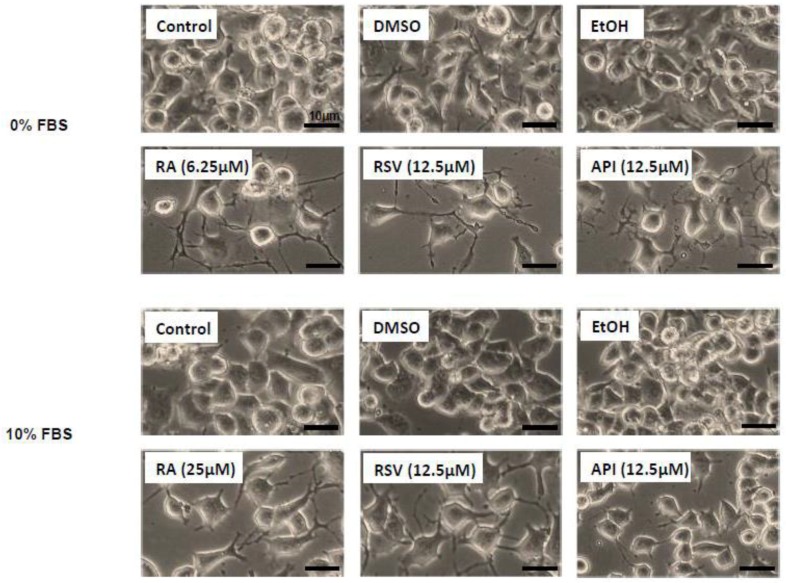
Morphological evaluation of neuronal differentiation of neuro-2a (N2a) cells treated with resveratrol, apigenin, and retinoic acid used as positive control. Murine neuronal N2a cells previously cultured for 24 h in conventional cultured medium were further cultured for 48 h in medium without or with 10% fetal bovine serum (FBS) in the absence or in the presence of retinoic acid (RA: 6.25 and 25 µM) respectively, used as positive control for the induction of neuronal differentiation, or with polyphenols: resveratrol (RSV 12.5 µM) and apigenin (API 12.5 µM). Control cells were cultured in medium without or with 10% FBS. Two vehicle controls were realized: ethanol (EtOH) used with RSV, and dimethyl sulfoxide (DMSO) used with RA and API. Observations were realized by phase contrast microscopy. Differentiated cells, characterized by neurite outgrowth (dendrites and/or axons), are observed in the presence of RSV, API, and RA.

**Figure 2:**
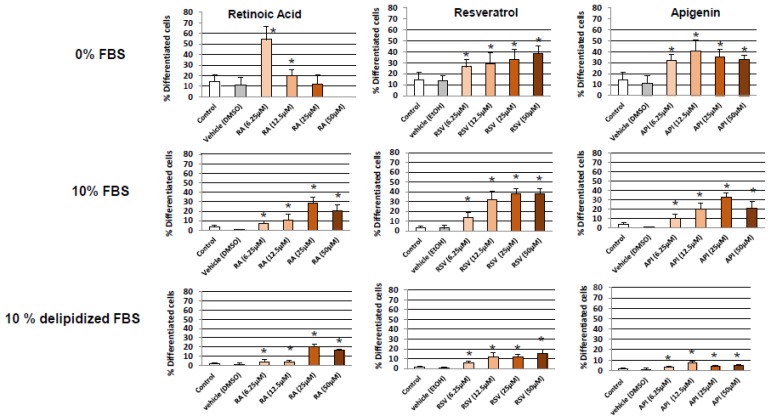
Quantification of neuronal differentiation induced by resveratrol and apigenin on N2a cells. Murine neuronal N2a cells previously cultured for 24 h in conventional cultured medium were further cultured for 48 h in medium without or with 10% FBS in the absence or in the presence of retinoic acid (RA: 6.25–50 µM) used as positive control for the induction of neuronal differentiation, or with polyphenols: resveratrol (RSV: 6.25–50 µM) and apigenin (API: 6.25–50 µM). Control cells were cultured in medium without and with 10% FBS. Two vehicle controls were realized: EtOH used with RSV, and DMSO used with RA and API. The percentages of differentiated cells include cells with dendrites, axons and dendrites + axons; these percentages were determined by phase contrast microscopy. Each value is the mean ± standard deviation (SD) of four independent experiments. An ANOVA test followed by a Mann-Whitney test were used. *p* values of 0.05 or less were considered as statistically significant. * comparison between control (untreated cells), vehicles (DMSO, Ethanol (EtOH)), and RSV, API and RA; no difference between control/vehicles (DMSO, EtOH) was observed.

N2a cells were previously cultured for 24 h in the following culture medium: DMEM high glucose (4.5 g/L) with stable glutamine and sodium pyruvate containing 10% FBS. They were further cultured for 48 h in DMEM with high glucose and stable glutamine and sodium pyruvate without FBS (0% FBS), or with 10% FBS in the absence or in the presence of RSV (6.25–50 µM) or API (6.25–50 µM). Ethanol (EtOH) was used as vehicle to dissolve RSV whereas DMSO was used as vehicle to dissolve API and RA (6.25–50 µM) used as positive control to induce neuronal differentiation. Neuronal differentiation induced by RSV, API, and RA was morphologically evaluated by phase contrast microscopy on 20 images taken with a Zeiss camera under an inverted Zeiss microscope (Figure 1). In those conditions, cell differentiation was evaluated by neurite outgrowth (dendrites and/or axons) and differentiated cells with dendrites, axons, and dendrites + axons were quantified. In the different conditions of culture, the spontaneous level of total differentiated cells (cells with dendrites, axons, and dendrites + axons) was similar with 0% FBS and with 10% FBS (Figure 2).

**Figure 3:**
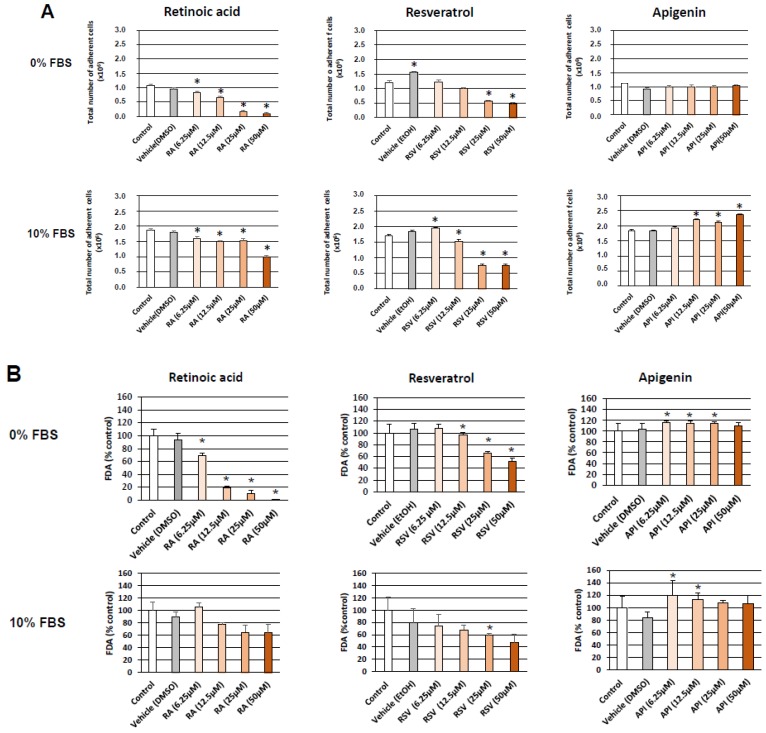
Incidence of neuronal differentiation induced by resveratrol and apigenin on cell growth and viability of N2a cells. Murine neuronal N2a cells previously cultured for 24 h in conventional cultured medium were further cultured for 48 h in medium without and with 10% FBS in the absence or in the presence of retinoic acid (RA: 6.25–50 µM) used as positive control for the induction of neuronal differentiation, or with polyphenols: resveratrol (RSV: 6.25–50 µM) and apigenin (API: 6.25–50 µM). Control cells were cultured in medium without and with 10% FBS. Two vehicle controls were realized: EtOH used with RSV, and DMSO used with RA and API. Cell growth (total number of adherent cells) was determined by cell counting (**A**) and cell viability by fluorimetry with the FDA test (**B**). Each value is the mean ± standard deviation (SD) of four independent experiments. An ANOVA test followed by a Mann–Whitney test were used. *p* values of 0.05 or less were considered as statistically significant. * comparison between control (untreated cells), vehicles (DMSO, EtOH), and RSV, API and RA; no difference between control/vehicles (DMSO, EtOH) was observed.

Without FBS (0% FBS), the highest percentage of differentiated cells was observed with RA (6.25 µM; 53 ± 9%) (Figure 2); at the highest concentrations, lower percentages of differentiated cells were detected (Figure 2): this could be due to a dose-dependent decrease of cell growth and/or viability induced by RA revealed by cell counting and staining with fluorescein diacetate (FDA), respectively (Figure 3A,B). In the presence of RSV and API, the neuronal differentiation was in the same range of order (28–40%) from 6.25 to 50 µM (Figure 2); at these concentrations, cell growth and cell viability was sometimes slightly but significantly increased with API (Figure 3A,B) whereas a significant decrease in cell growth and viability was found with RSV (6.25 and 12.5 µM) (Figure 3A,B).

Interestingly, with 10% FBS, the highest percentages of differentiated cells (30–38%) associated with a decrease of cell growth and viability were observed with RSV in a range of concentration from 12.5 to 50 µM (Figure 2 and Figure 3A,B). Similar differentiating effects (10–30%), in a range of concentration from 12.5 to 50 µM, without or with a slight cytotoxicity, were observed with API and RA (Figure 2 and Figure 3A,B).

Under treatment with RSV, and especially with API and RA, as the induction of differentiation, as well as the impact on cell growth and viability were different without FBS and with 10% FBS (the differentiation was often reduced in the presence of 10% FBS), N2a cells were treated with RSV, API, and RA in the presence of 10% delipidized FBS to determine the incidence of the lipidic and non-lipidic fraction of the serum on differentiation. Of note, in the presence of 10% delipidized FBS, the differentiating effects of RSV, API, and RA were strongly reduced, supporting that the inhibiting effects of the FBS on neuronal differentiation are due to non-lipidic compounds (Figure 2).

Preliminary data on the repartition of the cells in the different phases of the cell cycle have been obtained (Supplementary Figure S2A,B). Without FBS (0% FBS), slight modifications of the cell cycle were observed compared to the control and vehicle-treated cells in the presence of RSV, API, and RA used at a concentration inducing differentiation. With 10% FBS at concentrations inducing differentiation, modifications of the cell cycle were mainly observed with RSV (12.5 µM). However, at a concentration of 25–50 µM, which is used to induce cell death (especially the 50-µM concentration), RSV and API induce modifications of the cell cycle as previously reported. Thus, RSV (25–50 µM), in agreement with other studies [51,52], induces an accumulation of the cells in the S phase. API (25–50 µM) triggers a less pronounced accumulation of the cells in the S phase than that observed with RSV, whereas its ability to favor an accumulation in G2 + M has been reported [53,54]. RA induces an accumulation of the cells in G2 + M.

**Figure 4:**
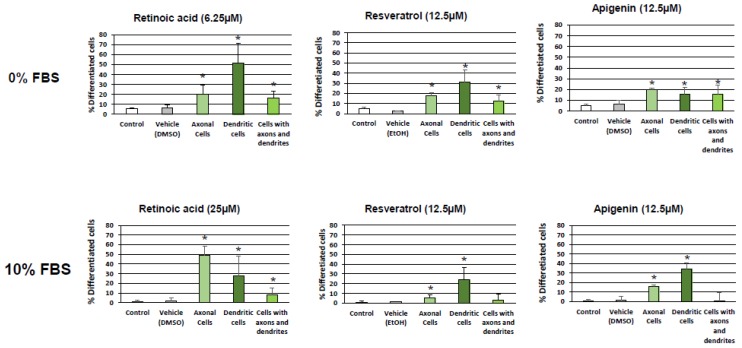
Characterization and quantification of differentiated neuronal cells (cells with dendrites, axons and dendrites + axons) induced by resveratrol and apigenin. Murine neuronal N2a cells previously cultured for 24 h in conventional cultured medium were further cultured for 48 h in medium without and with 10% FBS in the absence or in the presence of retinoic acid (RA: 6.25 µM with 0% FBS; 25 µM with 10% FBS) used as positive control for the induction of neuronal differentiation, or with polyphenols: resveratrol (RSV: 12.5 µM with 0% and 10% FBS) and apigenin (API: 12.5 µM with 0% and 10% FBS). Control cells were cultured in medium without and with 10% FBS. Two vehicle controls were realized: EtOH used with RSV, and DMSO used with RA and API. The percentages of cells with dendrites, axons, and axons + dendrites are shown, and were determined by phase contrast microscopy. Each value is the mean ± standard deviation (SD) of four independent experiments. An ANOVA test followed by a Mann–Whitney test were used. *p* values of 0.05 or less were considered as statistically significant. * comparison between control (untreated cells), vehicles (DMSO, EtOH), and RSV, API, and RA; no difference between control/vehicles (DMSO, EtOH) was observed.

For further experiments without FBS and with 10% FBS, the concentrations of RSV, API, and RA that were chosen are a compromise in terms of the ability to induce high differentiation without or with a low toxicity. The lowest concentrations meeting these criteria were selected: RSV and API, 12.5 µM without and with 10% FBS; and RA, 6.25 and 25 µM, without and with 10% FBS, respectively. In those conditions, RSV and API induce neurite outgrowth, and N2a cells with dendrites, axons, and dendrites + axons were observed (Figure 4). Depending on the compound considered the percentage of cells with dendrites or axons was more or less different without or with 10% FBS (Figure 4). The highest percentage of axonal cells (20–50%) which can be considered as a sign of terminal neuronal differentiation was observed with RA, whereas the percentage of axonal cells was from 22 to 30% and 16 to 34% with RSV and API, respectively (Figure 4; Supplementary Figure S3).

Altogether, these data show that RSV and API are potent inducers of neuronal differentiation, inducing neuritis and axon outgrowth (terminal neuronal differentiation). Of note, at the opposite of RA and RSV, these differentiating effects of API were associated with no or slight cytotoxic effects as shown by cell counting and the FDA test.

### 3.2. Characterization of Resveratrol- and Apigenin-Signaling Pathways Involved in the Neuronal Differentiation of N2a Cells

At the moment, little is known about the signaling pathways involved in the neuronal differentiation induced by neurotrophins. As octadecaneuropeptide (ODN), which can be considered as a neurotrophin, has cytoprotective effects via metabotropic receptors involving both the activation of the PKA/(PLC/PKC)/(MEK/ERK) signaling pathway [50], we determined whether PKA, PLC, PKC and MEK/ERK were involved in the neuronal differentiation induced by RSV and API (as well as of RA used as positive control) with different inhibitors: H89 (PKA), U0126 (MEK), U73122 (PLC) and chelerythrine (PKC).

**Figure 5:**
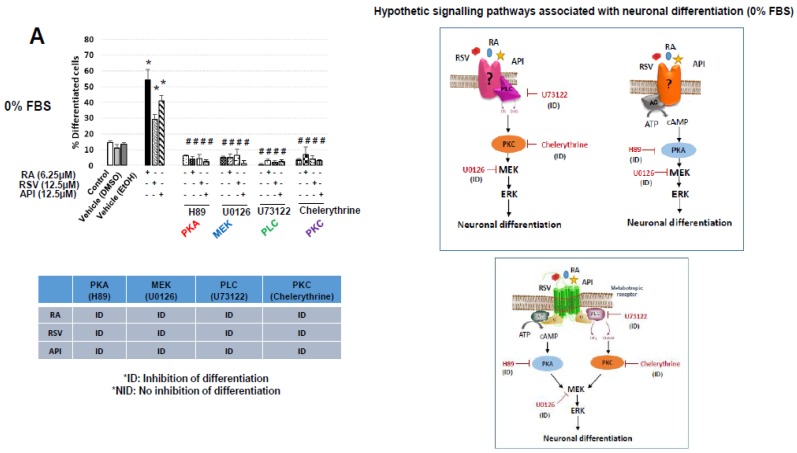

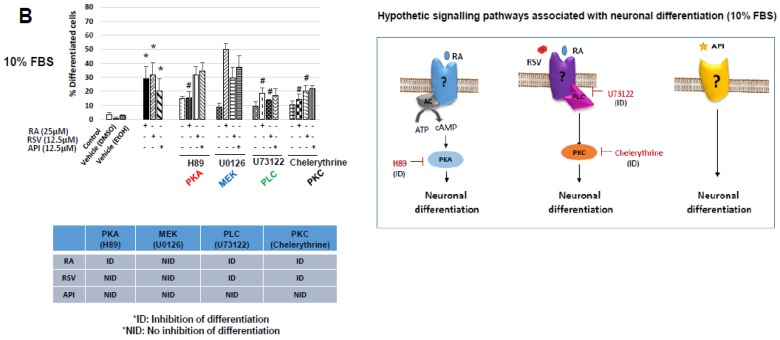
Evaluation of protein kinase A (PKA), phospholipase C (PLC)/protein kinase C (PKC) and the MEK/ERK signaling pathways in resveratrol- and apigenin-induced neuronal differentiation of N2a cells cultured without FBS (0% FBS) and with 10% FBS. Murine neuronal N2a cells previously cultured for 24 h in conventional cultured medium were further cultured for 48 h in medium without FBS (0% FBS) (**A**) or with 10% FBS (**B**) in the absence or in the presence of retinoic acid (RA: 6.25, 25 µM) respectively, used as positive control for the induction of neuronal differentiation, or with polyphenols: resveratrol (RSV: 12.5 µM) and apigenin (API: 12.5 µM). RSV and API were used either without or with different inhibitors: H89 (2 × 10^−5^ M; PKA), U0126 (10^−6^ M; MEK), U73122 (10^−7^ M; PLC) and chelerythrine (10^−7^ M; PKC) [50]. Control cells were cultured in medium without FBS (0% FBS) or with 10% FBS. Two vehicle controls were realized: EtOH used with RSV, and DMSO used with RA and API. Control cells and vehicle-treated cells were also cultured without or with H89, U0126, U73122 and chelerythrine. The percentages of differentiated cells were determined by phase contrast microscopy. The hypothetic signaling pathways associated with RSV, API, and RA without FBS (0% FBS) and with 10% FBS are represented on the right of the corresponding Figures and Tables. The values are means ± standard deviation (SD) of four independent experiments. An ANOVA test followed by a Mann–Whitney test were used. *p* values of 0.05 or less were considered as statistically significant. * comparison between control (untreated cells), vehicles (DMSO, EtOH), and RSV, API, and RA; # comparison between RSV, API, and RA, and RSV, API, and RA associated with H89, U0126, U73122, or chelerythrine.

Without FBS (0% FBS), when RSV and API were associated with H89, U0126, U73122, and chelerythrine, a total inhibition of neuronal differentiation (% of total differentiated cells: cells with dendrites and/or axons) was observed (Figure 5A). This supports that these two polyphenols simultaneously stimulate the following signaling pathways PLC/PKC/(MEK/ERK) and PKA/MEK, or that they act via a receptor evocating the canonical metabotropic receptors which simultaneously involves the activation of PKA and PLC/PKC, leading to activation of MEK/ERK (Figure 5A).

With 10% FBS, important modifications in the signaling pathways activated by RSV and API were observed. With RSV, PLC/PKC as well as PKA were required for the induction of neuronal differentiation (Figure 5B). With API, the neuronal differentiation was independent of PKA, PLC/PKC, and MEK/ERK (Figure 5B). With retinoic acid, PLC/PKC were only required for the induction of neuronal differentiation (Figure 5B). Thus, with RSV, PLC/PKC were activated both with 0% and 10% FBS; with API, PKA, PLC/PKC, and MEK/ERK were activated with 0% FBS, whereas none of these pathways were needed with 10% FBS; RA, PKA, and PLC/PKC were activated both with 0% and 10% FBS (Figure 5A,B).

### 3.3. Evaluation of the Involvement of Octadecaneuropeptide (ODN) Receptor in the Neuronal Differentiation of N2a Cells Induced by Resveratrol and Apigenin

**Figure 6:**
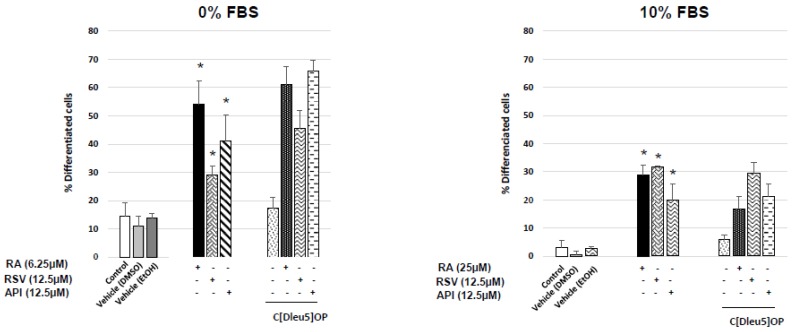
Evaluation of the implication of the ODN receptor in neuronal differentiation induced by resveratrol and apigenin on N2a cells. Murine neuronal N2a cells previously cultured for 24 h in conventional cultured medium were further cultured for 48 h in medium without or with 10% FBS in the absence or in the presence of retinoic acid (RA: 6.25 µM with 0% FBS; 25 µM with 10% FBS) used as positive control for the induction of neuronal differentiation, or with polyphenols: resveratrol (RSV: 12.5 µM with 0% and 10% FBS) and apigenin (API: 12.5 µM with 0% and 10% FBS). RSV and API were cultured either without or with Cyclo_1–5_[Dleu^5^] OP (10^−6^ M; an antagonist of ODN metabotropic receptor) [50]. Control cells were cultured in medium without and with 10% FBS. Two vehicle controls were realized: EtOH used with RSV, and DMSO used with RA and API. Control cells and vehicle-treated cells were also cultured without or with Cyclo_1–5_[Dleu^5^] OP. The percentages of differentiated cells were determined by phase contrast microscopy. The values are means ± standard deviation (SD) of four independent experiments. An ANOVA test followed by a Mann–Whitney test were used. *p* values of 0.05 or less were considered as statistically significant. * comparison between control (untreated cells), vehicles (DMSO, EtOH), and RSV, API, and RA; no significant differences were observed between RSV, API and RA, and RSV, API, and RA associated with C[Dleu^5^] OP. No difference was observed between control/vehicles (DMSO, EtOH) or between control/C[Dleu^5^] OP.

Based on the data obtained with RSV and API without FBS (0% FBS), an involvement of the metabotropic receptors of ODN can be suspected. We therefore simultaneously cultured N2a cells with RSV and API, without and with cyclo_1–8_[DLeu^5^] OP (an antagonist ODN metabotropic receptor) [50]. In those conditions, the neuronal differentiation induced by RSV and API was not reduced (Figure 6) supporting that the direct activity of RSV and API does not require the metabotropic receptors of ODN, or that RSV and API do not activate the synthesis of DBI, the precursor of ODN [37]. In agreement with this later hypothesis, in comparison to untreated and vehicle-treated cells, no increase of DBI mRNA levels evaluated with the Ct values were observed by RT-qPCR when N2a cells were cultured with resveratrol and apigenin; similar data were found with retinoic acid (Table 1).

In addition, under treatment with RSV and API, no increase of nerve growth factor (NGF) and brain-derived neurotrophic factor (BDNF) mRNA levels evaluated with the Ct values was observed by RT-qPCR (Table 1). Similarly, no effect of RA, on DBI, NGF, and BDNF mRNA level was found (Table 1).

## 4. Discussion

Polyphenols are a broad family of molecules including flavonoids, phenolic acids, lignans and stilbenes (such as resveratrol) [3,4]. Flavonoids include flavones (such as apigenin), flavonols, flavanones, flavanols, isoflavones, and anthocyanins. The polyphenols are found in large quantities in the Mediterranean diet which is rich in fruits and vegetables and which can be associated with a consumption of wine [55]. Polyphenols are for the most part powerful antioxidants, and some of them, such as apigenin and resveratrol, also have anti-proliferative and anti-inflammatory properties and decrease the production of growth factors as insulin growth factor-1 (IGF-1) and vascular endothelial growth factor (VEGF) [15,56]. With resveratrol, pro-differentiating activities have also been shown on myoblasts, resulting in the formation of myotubes [57]. Apigenin has also been shown to promote osteogenic differentiation of human mesenchymal stem cells through JNK and p38 MAPK pathways [58,59], to enhance myoblast differentiation by regulating Prmt7 [60], to induce granulocytic differentiation in human promyelocytic leukemia HL-60 cells [61], and to activate morphological differentiation and G2-M arrest in rat neuronal cells [62]. It has also been reported that apigenin from Croton betulaster Mull inhibits proliferation, induces differentiation and regulates the inflammatory profile of C6 glioma cells [63].

In response to the increase in age-related diseases, particularly neurodegenerative diseases associated with oxidative stress and inflammation [5], polyphenols, because of their anti-oxidant and anti-inflammatory activities, could be used for prevention or even as a treatment. Several studies also reveal a benefit of polyphenols to cognitive functions [22]. Moreover, it is now well established that resveratrol prevents the aggregation of β-amyloid, which is neurotoxic [64]. In the more common neurodegenerative diseases such as Alzheimer’s disease and Parkinson’s disease, neuronal loss is associated with the evolution of these diseases. Promoting neurogenesis could be a means of preventing these diseases. In this context, neuropeptides produced by the brain via endothelial cells and astrocytes form a family of molecules called neurotrophins, with cytoprotective and differentiating activities [65]. However, to exert their cytoprotective effects, these molecules must be injected intracerebrally near the lesions [66]. As polyphenols have the ability to cross the blood–brain barrier and accumulate in the brain much more efficiently when administered intravenously than orally [27,67,68], these properties reinforce their interest in preventing certain neurodegenerative diseases.

In this context, it was therefore important to clarify whether some polyphenols (resveratrol, apigenin) present in significant amount in the Mediterranean diet could promote neurogenesis, especially the differentiation of neuroblasts into mature neurons characterized by the presence of dendrites and/or axons. Using N2a murine neuroblastoma cells, we demonstrated that these cells cultured in the presence of resveratrol and apigenin in a concentration range from 6.25 to 50 μM differentiate into mature neurons with dendrites and/or axons and that the activation of the PKC signaling pathway plays an important role in this process. These results, which establish that some polyphenols (resveratrol, apigenin) are able to trigger neuronal differentiation, opening new perspectives in terms of treatments for neurodegenerative diseases where only few molecules are effective.

Interestingly, the differentiation observed with resveratrol and apigenin on N2a cells is as effective as that obtained with retinoic acid (used as positive control), which in humans can be associated with various side effects because of its ability to activate or suppress the expression of many genes [69]. In the present study, in terms of toxicity, the differentiation induced by resveratrol as well as with retinoic acid is associated with an inhibition of cell growth resulting from a decrease of cell viability, as evaluated with the FDA test. On the other hand, with apigenin, neuronal differentiation has only little effect on cell growth and viability, suggesting different mechanisms of differentiation between resveratrol and apigenin. In addition, the differentiation observed in the absence of FBS (0% FBS) demonstrates that exogenous factors present in the serum do not contribute to the neuronal differentiation induced by resveratrol and apigenin as well as with retinoic acid. Moreover, a comparison of the efficiency of resveratrol and apigenin to induce neuronal differentiation in serum-free culture medium (0% FBS) and in culture medium containing 10% FBS clearly shows that FBS attenuates the differentiation induced by the polyphenols and retinoic acid, and that FBS also contributes to the attenuation of the cytotoxicity, mainly with retinoic acid. As the comparison of the differentiation obtained in the presence of delipidized serum (10% delipidized FBS) versus 10% FBS shows a lower differentiation in the presence of 10% delipidized FBS than in the presence of 10% FBS, our results demonstrate that some serum proteins counteract neuronal differentiation. The identification of these molecules could make it possible to optimize the use of polyphenols for neurodifferentiation purposes in humans. The fractionation of the serum associated with a proteomic analysis must make it possible to answer this question. It has been reported that serum factors such as α_1_-, α_4_-, and β-globulin fractions can cause the inhibition of neuronal differentiation and neurite growth [70,71,72], whereas serum deprivation increases the phosphorylation of EGFR, ERK1/2, AKt, and other signaling molecules in N2a cells [72]. On the basis of in vitro tests using patient serum instead of FBS, one can also expect to distinguish between good and bad responders to treatment with polyphenols.

In addition, as the ability of RSV, API, and RA to trigger neuronal differentiation occurs without and with 10% FBS, and is associated with modifications of the cell cycle (RSV and API: marked and slight accumulation of the cells in the S phase of the cell cycle, respectively; RA: accumulation of the cells in the G2 + M phase of the cell cycle), the role taken by cell cycle-associated molecules in the neuronal differentiation of N2a cells will require additional investigation.

Altogether, these data underline that in order to promote neurogenesis with polyphenols, it is necessary to identify the signaling pathways involved in this process to develop effective drugs and to optimize the efficiency of resveratrol and apigenin. Hence, we tried to specify which signaling pathways were activated with resveratrol and apigenin but also with retinoic acid used as a positive control.

Due to the ability of certain neurotrophic factors (characterized by cytoprotective and differentiating properties) such as ODN to exert their activities via metabotropic receptors (G protein-coupled receptors) [73], some molecules capable of inhibiting the metabotropic receptor-associated response under the effect of ODN were used: H89 (PKA inhibitor), U0126 (MEK inhibitor), U73122 (PLC inhibitor), and chelerythrine (PKC inhibitor) [38,50]. Without FBS, when RSV and API were associated with H89, U0126, U73122, and chelerythrine, a total inhibition of neuronal differentiation was observed supporting either that these two polyphenols are able to simultaneously stimulate the PLC/PKC/(MEK/ERK) and PKA/(MEK/ERK) signaling pathways, or that they act via a receptor evocating the metabotropic receptors which simultaneously involves the activation of PKA and PLC/PKC, leading to the activation of MEK/ERK. However, with 10% FBS, important modifications in the signaling pathways activated by RSV and API were observed. With RSV, PLC/PKC as well as PKA were activated, whereas with API, the neuronal differentiation was independent of PKA, PLC/PKC, and MEK/ERK. Thus, with RSV, the PLC/PKC signaling pathway was activated both with 0% and 10% FBS, whereas with API, PKA, PLC/PKC, and MEK/ERK were activated with 0% FBS. None of these pathways were required with 10% FBS. These data bring new evidence supporting that the biological activities of RSV and API (impact on cell growth, viability, and differentiation) involve different mechanisms. With RA (used as positive control), PKA and PLC/PKC were activated both with 0% and 10% FBS. Altogether, these data support that the early metabolic pathways involved in neuronal differentiation depend on the inducer considered, and that the differentiating activities of polyphenols (RSV, API) could involve plasma membrane receptors.

In the absence of serum, as the implication of a receptor evoking the metabotropic receptor and simultaneously activating the PKC and PKA pathways was considered [73,74], we attempted to determine whether RSV and API were able to activate the synthesis of DBI, which is an ODN precursor [37]. In those conditions, the ODN produced could in turn activate the metabotropic receptor in an autocrine or paracrine way. The very low values of Ct obtained for DBI in the control cells as well as in the cells cultured in the absence or presence of RSV and API, without FBS or with 10% FBS, excludes this hypothesis. In addition, no effects of RSV and API were observed on NGF and BDNF mRNA levels.

As RSV and API display more ability to differentiate N2a cells than RA, we suggest the following potential mechanism behind this phenomenon. The growth of dendrites and/or neurons requires much energy and mitochondrial biogenesis [75]. In addition, dietary micronutrients (including polyphenols) have been shown to favor mitochondrial/nuclear dialogue, which could favor gene expression such as in those involved in neuronal differentiation [76]. In well-differentiated neuronal cells, a topographical redistribution of mitochondria along the axone is also needed to favor the transmission of the nerve impulse [77]. As several polyphenols are recognized as molecules capable of modulating pathways involved in mitochondrial biogenesis (induction of sirtuins), mitochondrial activity (modulating complexes I to V activity, ATP production), and control of the intra-mitochondrial oxidative status (inhibition of ROS formation), the different benefits of this family of compounds (including RSV and API) at the mitochondrial level might be predominant [78]. In addition, the mitochondria also play key roles in lipid metabolism, especially fatty acids, which are required for the biogenesis of lipids (fatty acids, phospholipids) present in the membrane of dendrites and neurons [79]. In contrast, RA is rather known to favor mitochondrial permeability and transition, leading to apoptosis [80]. It is therefore suggested that the different impacts of RSV, API, and RA at the mitochondrial level, and also probably in other organelles (lysosome, peroxisome) playing key roles in the control of lipid metabolism and the equilibrium between life and death might contribute, at least in part, to the different neuronal differentiation capacities of these different molecules.

## 5. Conclusions

Our data obtained on murine neuroblastoma N2a cells establish that RSV and API are able to induce neuronal differentiation and favor neurogenesis characterized by neurite outgrowth. Thus, when treated with RSV and API, the differentiated N2a cells are characterized by the presence of dendrites and axons and less frequently by the simultaneous presence of dendrites + axons. These morphological criteria demonstrate that RSV and API trigger a neuronal differentiation inducing the formation of mature neurons. Since polyphenols also exhibit cytoprotective, mainly anti-oxidant properties on neurodegeneration models [81,82], their cytoprotective and neuron-differentiating properties suggest that these compounds may mimic neurotrophin activities. These data support that micronutrients such as RSV and API, which are widely represented in the Mediterranean diet, could be of interest for the prevention and/or the treatment of neurodegenerative diseases associated with neuronal loss (Alzheimer’s disease, Parkinson’s disease). These data also reinforce the interest of polyphenols for the treatment of major aged-related diseases associated with neurodegeneration (Alzheimer’s disease, Parkinson’s disease) for which the therapeutic arsenal is reduced.

## Figures and Tables

**Table 1:** Effects of resveratrol, apigenin, and retinoic acid on the Ct values of neuropeptides with potential neurotrophin activities: diazepam binding inhibitor (DBI; the precursor of octadecaneuropeptide (ODN)), nerve growth factor (NGF) and brain derived neurotrophic factors (BDNF).

Neuropeptides	% FBS	Ct					
		Control	EtOH (0.02 %)	DMSO (0.12 %)	RA	RSV	API
**DBI**	**0 %**	33.5 ± 0.1	33.5 ± 1.6	30.6 ± 0.3	33.6 ± 1.0	33.4 ± 1.2	31.9 ± 0.1
	**10 %**	30.8 ± 0.5	28.4 ± 0.3	28.2 ± 0.3	32.2 ± 0.2	28.9 ± 2.4	28.1 ± 0.5
**NGF**	**0 %**	32.9 ± 3.7	31.3 ± 1.3	28.1 ± 0.6	29.7 ± 0.2	30.0 ± 0.3	28.5 ± 0.1
	**10 %**	27.8 ± 0.5	25.4 ± 1.6	25.6 ± 1.8	28.6 ± 0.2	24.9 ± 0.2	25.3 ± 0.2
**BDNF**	**0 %**	34.8 ± 0.8	33.4 ± 1.1	29.7 ± 1.1	32.6 ± 0.7	35.5 ± 2.3	31.3 ± 1.7
	**10 %**	31.0 ± 0.6	28.1 ± 0.2	27.4 ± 0.5	31.1 ± 0.5	37.6 ± 0.5*	27.6 ± 0.1

Ct values were obtained by RT-qPCR on N2a cells. N2a cells were previously cultured for 24 h in conventional cultured medium; they were further cultured for 48 h in medium without or with 10% FBS in the absence or in the presence of retinoic acid (RA: 6.25 µM with 0% FBS; 25 µM with 10% FBS) used as positive control for the induction of neuronal differentiation, or with polyphenols: resveratrol (RSV: 12.5 µM with 0% and 10% FBS) and apigenin (API: 12.5 µM with 0% and 10% FBS). Two vehicle controls were realized: ethanol (EtOH) used with RSV, DMSO used with RA and API. Data shown are the mean ± SD of two independent experiments realized in triplicate. With the Mann-Whitney test, for DBI, NGF and BDNF, no significant differences were observed between control, ETOH (0.02%) and DMSO (0.12%). For DBI and NGF, no significant differences were found between RA, RSV, API and the corresponding vehicles. For BDNF, no significant differences were found between RA, RSV, API and the corresponding vehicles, excepted with RSV (10% FBS; * *p* ≤ 0.05%).

## Supplementary Materials

The following are available online at http://www.mdpi.com/2079-9721/6/3/67/s1, Supplementary Figure S1: Morphological criteria allowing discrimination between cells with dendrites, axons, and dendrites + axons. Murine neuronal N2a cells previously cultured for 24 h were further cultured for 48 h in medium without FBS (0% FBS) or with 10% FBS in the presence of ODN (10^−14^ and 10^−12^ M) used as neuronal differentiation inducer. In the presence of ODN, N2a cells were differentiated in neurons with dendrites (D), axons (A), and dendrites + axons (A + D). These morphological criteria were used to quantify the neuronal differentiation induced by RSV, API, and RA. Supplementary Figure S2: Impact of resveratrol, apigenin and retinoic acid on the repartition of the cells in the different phases of the cell cycle when cultured without or with 10% fetal bovine serum. Murine neuronal N2a cells previously cultured for 24 h in conventional cultured medium were further cultured for 48 h in medium without or with 10% FBS in the absence or in the presence of RA (6.25, 12.5 and 25 µM) used as positive control for the induction of neuronal differentiation, or with polyphenols: RSV (12.5, 25 and 50 µM), and API (12.5, 25 and 50 µM). Cell cycle analysis was performed by flow cytometry after staining with propidium iodide. Preliminary data shown correspond to one experiment. The ability of RSV (25 and 50 µM) to increase the percentage of cells in the S phase of the cell cycle validates the experiment. A—Histograms obtained by flow cytometry illustrating the repartition of N2a in the different phases of the cell cycle under treatment with RSV, API, and RA in culture medium without or with 10% FBS; B—percentages of cells in the different phases of the cell cycle (N2a cells treated with RSV, API and RA in culture medium without or with 10% FBS. Supplementary Figure S3: Morphological aspect of N2a cells cultured without or with 10% FBS in the presence of retinoic acid, resveratrol, or apigenin. Murine neuronal N2a cells previously cultured for 24 h in conventional cultured medium were further cultured for 48 h in medium without and with 10% FBS in the absence or in the presence of RA (6.25 µM with 0% FBS; 25 µM with 10% FBS) used as positive control for the induction of neuronal differentiation, or with polyphenols: RSV (12.5 µM with 0% and 10% FBS) and API (12.5 µM with 0% and 10% FBS). Control cells were cultured in medium without and with 10% FBS. Two vehicle controls were realized: EtOH used with RSV, DMSO used with RA and API. The supplementary Figure S3 illustrates the data presented in Figure 4. The morphological aspect of N2a cells cultured without or with 10% FBS in the presence of retinoic acid, resveratrol, or apigenin is shown. The pictures were obtained by phase contrast microscopy.

## References

[1] Bravo L. (1998). Polyphenols: Chemistry, dietary sources, metabolism, and nutritional significance. Nutr. Rev..

[2] Manach C., Scalbert A., Morand C., Rémésy C., Jiménez L. (2004). Polyphenols: Food sources and bioavailability. Am. J. Clin. Nutr..

[3] Del Rio D., Rodriguez-Mateos A., Spencer J.P., Tognolini M., Borges G., Crozier A. (2013). Dietary (poly)phenolics in human health: Structures, bioavailability, and evidence of protective effects against chronic diseases. Antioxid. Redox Signal..

[4] Santhakumar A.B., Battino M., Alvarez-Suarez J.M. (2018). Dietary polyphenols: Structures, bioavailability and protective effects against atherosclerosis. Food Chem. Toxicol..

[5] Zarrouk A., Vejux A., Mackrill J., O’Callaghan Y., Hammami M., O’Brien N., Lizard G. (2014). Involvement of oxysterols in age-related diseases and ageing processes. Ageing Res. Rev..

[6] Cilla A., Alegría A., Attanzio A., Garcia-Llatas G., Tesoriere L., Livrea M.A. (2017). Dietary phytochemicals in the protection against oxysterol-induced damage. Chem. Phys. Lipids.

[7] Upadhyay S., Dixit M. (2015). Role of Polyphenols and Other Phytochemicals on Molecular Signaling. Oxid. Med. Cell. Longev..

[8] Carito V., Ceccanti M., Tarani L., Ferraguti G., Chaldakov G.N., Fiore M. (2016). Neurotrophins’modulation by olive polyphenols. Curr. Med. Chem..

[9] Tan S., Schubert D., Maher P. (2001). Oxytosis: A novel form of programmed cell death. Curr. Top. Med. Chem..

[10] Schaffer S., Eckert G.P., Schmitt-Schillig S., Müller W.E. (2006). Plant foods and brain aging: A critical appraisal. Forum Nutr..

[11] Farinetti A., Zurlo V., Manenti A., Coppi F., Mattioli A.V. (2017). Mediterranean diet and colorectal cancer: A systematic review. Nutrition.

[12] Gorzynik-Debicka M., Przychodzen P., Cappello F., Kuban-Jankowska A., Marino Gammazza A., Knap N., Wozniak M., Gorska-Ponikowska M. (2018). Potential Health Benefits of Olive Oil and Plant Polyphenols. Int. J. Mol. Sci..

[13] Kang H.J., Youn Y.K., Hong M.K., Kim L.S. (2011). Antiproliferation and redifferentiation in thyroid cancer cell lines by polyphenol phytochemicals. J. Korean Med. Sci..

[14] Kaminski J., Lançon A., Aires V., Limagne E., Tili E., Michaille J.J., Latruffe N. (2012). Resveratrol initiates differentiation of mouse skeletal muscle-derived C2C12 myoblasts. Biochem. Pharmacol..

[15] Latruffe N., Rifler J.P. (2013). Bioactive polyphenols from grapes and wine emphasized with resveratrol. Curr. Pharm. Des..

[16] Li H., Liu Y., Jiao Y., Guo A., Xu X., Qu X., Wang S., Zhao J., Li Y., Cao Y. (2016). Resveratrol sensitizes glioblastoma-initiating cells to temozolomide by inducing cell apoptosis and promoting differentiation. Oncol. Rep..

[17] Zhao L., Wang J.L., Liu R., Li X.X., Li J.F., Zhang L. (2013). Neuroprotective, anti-amyloidogenic and neurotrophic effects of apigenin in an Alzheimer’s disease mouse model. Molecules.

[18] Guo L., Wang L., Wang L., Yun-Peng S., Zhou J.J., Zhao Z., Li D.P. (2017). Resveratrol Induces Differentiation of Human Umbilical Cord Mesenchymal Stem Cells into Neuron-Like Cells. Stem Cells Int..

[19] Leonarduzzi G., Testa G., Sottero B., Gamba P., Poli G. (2010). Design and development of nanovehicle-based delivery systems for preventive or therapeutic supplementation with flavonoids. Curr. Med. Chem..

[20] Testa G., Gamba P., Badilli U., Gargiulo S., Maina M., Guina T., Calfapietra S., Biasi F., Cavalli R., Poli G. (2014). Loading into nanoparticles improves quercetin’s efficacy in preventing neuroinflammation induced by oxysterols. PLoS ONE.

[21] Sahni J.K., Doggui S., Ali J., Baboota S., Dao L., Ramassamy C. (2011). Neurotherapeutic applications of nanoparticles in Alzheimer’s disease. J. Control. Release.

[22] Poulose S.M., Miller M.G., Scott T., Shukitt-Hale B. (2017). Nutritional Factors Affecting Adult Neurogenesis and Cognitive Function. Adv. Nutr..

[23] Moosavi F., Hosseini R., Saso L., Firuzi O. (2015). Modulation of neurotrophic signaling pathways by polyphenols. Drug Des. Dev. Ther..

[24] Costa S.L., Silva V.D., Dos Santos Souza C., Santos C.C., Paris I., Muñoz P., Segura-Aguilar J. (2016). Impact of Plant-Derived Flavonoids on Neurodegenerative Diseases. Neurotox. Res..

[25] Ebadi M., Bashir R.M., Heidrick M.L., Hamada F.M., Refaey H.E., Hamed A., Helal G., Baxin M.D., Cerutis D.R., Lassi N.K. (1997). Neurotrophins and their receptors in nerve injury and repair. Neurochem. Int..

[26] Scalbert A., Morand C., Manach C., Rémésy C. (2002). Absorption and metabolism of polyphenols in the gut and impact on health. Biomed. Pharmacother..

[27] Figueira I., Garcia G., Pimpão R.C., Terrasso A.P., Costa I., Almeida A.F., Tavares L., Pais T.F., Pinto P., Ventura M.R. (2017). Polyphenols journey through blood-brain barrier towards neuronal protection. Sci. Rep..

[28] Akagi M., Matsui N., Akae H., Hirashima N., Fukuishi N., Fukuyama Y., Akagi R. (2015). Nonpeptide neurotrophic agents useful in the treatment of neurodegenerative diseases such as Alzheimer’s disease. J. Pharmacol. Sci..

[29] Zhang J.C., Xu H., Yuan Y., Chen J.Y., Zhang Y.J., Lin Y., Yuan S.Y. (2017). Delayed Treatment with green tea polyphenol egcg promotes neurogenesis after ischemic stroke in adult mice. Mol. Neurobiol..

[30] Dias G.P., Cocks G., do Nascimento Bevilaqua M.C., Nardi A.E., Thuret S. (2016). Resveratrol: A Potential Hippocampal Plasticity Enhancer. Oxid. Med. Cell. Longev..

[31] Farzaei M.H., Abbasabadi Z., Ardekani M.R., Rahimi R., Farzaei F. (2013). Parsley: A review of ethnopharmacology, phytochemistry and biological activities. J. Tradit. Chin. Med..

[32] Khalil M.I., Sulaiman S.A., Boukraa L. (2010). Antioxidant properties of honey and its role in preventing health disorders. Open Nutraceut. J..

[33] Ricciutelli M., Marconi S., Boarelli M.C., Caprioli G., Sagratini G., Ballini R., Fiorini D. (2017). Olive oil polyphenols: A quantitative method by high-performance liquid-chromatography-diode-array detection for their determination and the assessment of the related health claim. J. Chromatogr. A.

[34] Losi G., Puia G., Garzon G., Vuono M.C., Baraldi M. (2004). Apigenin modulates GABAergic and glutamatergic transmission in cultured cortical neurons. Eur. J. Pharmacol..

[35] Kojima N., Kurosawa N., Nishi T., Hanai N., Tsuji S. (1994). Induction of cholinergic differentiation with neurite sprouting by de novo biosynthesis and expression of GD3 and b-series gangliosides in Neuro2a cells. J. Biol. Chem..

[36] Tremblay R.G., Sikorska M., Sandhu J.K., Lanthier P., Ribecco-Lutkiewicz M., Bani-Yaghoub M. (2010). Differentiation of mouse Neuro 2A cells into dopamine neurons. J. Neurosci. Methods.

[37] Costa E., Guidotti A. (1991). Diazepam binding inhibitor (DBI): A peptide with multiple biological actions. Life Sci..

[38] Hamdi Y., Kaddour H., Vaudry D., Bahdoudi S., Douiri S., Leprince J., Castel H., Vaudry H., Tonon M.C., Amri M. (2012). The octadecaneuropeptide ODN protects astrocytes against hydrogen peroxide-induced apoptosis via a PKA/MAPK-dependent mechanism. PLoS ONE.

[39] Dickson L., Finlayson K. (2009). VPAC and PAC receptors: From ligands to function. Pharmacol. Ther..

[40] Vaudry D., Falluel-Morel A., Bourgault S., Basille M., Burel D., Wurtz O., Fournier A., Chow B.K., Hashimoto H., Galas L. (2009). Pituitary adenylate cyclase activating polypeptide and its receptors: 20 years after the discovery. Pharmacol. Rev..

[41] Monaghan T.K., Mackenzie C.J., Plevin R., Lutz E.M. (2008). PACAP-38 induces neuronal differentiation of human SH-SY5Y neuroblastoma cells via cAMP-mediated activation of ERK and p38 MAP kinases. J. Neurochem..

[42] May V., Lutz E., MacKenzie C., Schutz K.C., Dozark K., Braas K.M. (2010). Pituitary adenylate cyclase-activating polypeptide (PACAP)/PAC1HOP1 receptor activation coordinates multiple neurotrophic signaling pathways: Akt activation through phosphatidylinositol 3-kinase gamma and vesicle endocytosis for neuronal survival. J. Biol. Chem..

[43] Castorina A., Scuderi S., D’Amico A.G., Drago F., D’Agata V. (2014). PACAP and VIP increase the expression of myelin-related proteins in rat schwannoma cells: Involvement of PAC1/VPAC2 receptor-mediated activation of PI3K/Akt signaling pathways. Exp. Cell Res..

[44] Hirabayashi T., Nakamachi T., Shioda S. (2018). Discovery of PACAP and its receptors in the brain. J. Headache Pain.

[45] Dasgupta B., Milbrandt J. (2007). Resveratrol stimulates AMP kinase activity in neurons. Proc. Natl. Acad. Sci. USA.

[46] Marzinke M.A., Clagett-Dame M. (2012). The all-trans retinoic acid (atRA)-regulated gene Calmin (Clmn) regulates cell cycle exit and neurite outgrowth in murine neuroblastoma (Neuro2a) cells. Exp. Cell Res..

[47] Gonzalez B.J., Basille M., Vaudry D., Fournier A., Vaudry H. (1997). Pituitary adenylate cyclase-activating polypeptide promotes cell survival and neurite outgrowth in rat cerebellar neuroblasts. Neuroscience.

[48] Botia B., Basille M., Allais A., Raoult E., Falluel-Morel A., Galas L., Jolivel V., Wurtz O., Komuro H., Fournier A. (2007). Neurotrophic effects of PACAP in the cerebellar cortex. Peptides.

[49] Ogata K., Shintani N., Hayata-Takano A., Kamo T., Higashi S., Seiriki K., Momosaki H., Vaudry D., Vaudry H., Galas L. (2015). PACAP enhances axon outgrowth in cultured hippocampal neurons to a comparable extent as BDNF. PLoS ONE.

[50] Kaddour H., Hamdi Y., Vaudry D., Basille M., Desrues L., Leprince J., Castel H., Vaudry H., Tonon M.C., Amri M. (2013). The octadecaneuropeptide ODN prevents 6-hydroxydopamine-induced apoptosis of cerebellar granule neurons through a PKC-MAPK-dependent pathway. J. Neurochem..

[51] Marel A.K., Lizard G., Izard J.C., Latruffe N., Delmas D. (2008). Inhibitory effects of trans-resveratrol analogs molecules on the proliferation and the cell cycle progression of human colon tumoral cells. Mol. Nutr. Food Res..

[52] Colin D., Lancon A., Delmas D., Lizard G., Abrossinow J., Kahn E., Jannin B., Latruffe N. (2008). Antiproliferative activities of resveratrol and related compounds in human hepatocyte derived HepG2 cells are associated with biochemical cell disturbance revealed by fluorescence analyses. Biochimie.

[53] Wang W., Heideman L., Chung C.S., Pelling J.C., Koehler K.J., Birt D.F. (2000). Cell-cycle arrest at G2/M and growth inhibition by apigenin in human colon carcinoma cell lines. Mol. Carcinog..

[54] Elsisi N.S., Darling-Reed S., Lee E.Y., Oriaku E.T., Soliman K.F. (2005). Ibuprofen and apigenin induce apoptosis and cell cycle arrest in activated microglia. Neurosci. Lett..

[55] Latruffe N. (2017). Vin, Nutrition Méditerranéenne et Santé: Une Association Vertueuse.

[56] Dugas B., Charbonnier S., Baarine M., Ragot K., Delmas D., Ménétrier F., Lherminier J., Malvitte L., Khalfaoui T., Bron A. (2010). Effects of oxysterols on cell viability, inflammatory cytokines, VEGF, and reactive oxygen species production on human retinal cells: Cytoprotective effects and prevention of VEGF secretion by resveratrol. Eur. J. Nutr..

[57] Lançon A., Michaille J.J., Latruffe N. (2013). Effects of dietary phytophenols on the expression of microRNAs involved in mammalian cell homeostasis. J. Sci. Food Agric..

[58] Zhang X., Zhou C., Zha X., Xu Z., Li L., Liu Y., Xu L., Cui L., Xu D., Zhu B. (2015). Apigenin promotes osteogenic differentiation of human mesenchymal stem cells through JNK and p38 MAPK pathways. Mol. Cell. Biochem..

[59] Melguizo-Rodríguez L., Manzano-Moreno F.J., De Luna-Bertos E., Rivas A., Ramos-Torrecillas J., Ruiz C., García-Martínez O. (2018). Effect of olive oil phenolic compounds on osteoblast differentiation. Eur. J. Clin. Investig..

[60] Jang Y.J., Son H.J., Choi Y.M., Ahn J., Jung C.H., Ha T.Y. (2017). Apigenin enhances skeletal muscle hypertrophy and myoblast differentiation by regulating Prmt7. Oncotarget.

[61] Nakazaki E., Tsolmon S., Han J., Isoda H. (2013). Proteomic study of granulocytic differentiation induced by apigenin 7-glucoside in human promyelocytic leukemia HL-60 cells. Eur. J. Nutr..

[62] Sato F., Matsukawa Y., Matsumoto K., Nishino H., Sakai T. (1994). Apigenin induces morphological differentiation and G2-M arrest in rat neuronal cells. Biochem. Biophys. Res. Commun..

[63] Coelho P.L., Oliveira M.N., da Silva A.B., Pitanga B.P., Silva V.D., Faria G.P., Sampaio G.P., Costa M.F., Braga-de-Souza S., Costa S.L. (2016). The flavonoid apigenin from Croton betulaster Mull inhibits proliferation, induces differentiation and regulates the inflammatory profile of glioma cells. Anticancer Drugs.

[64] Jia Y., Wang N., Liu X. (2017). Resveratrol and Amyloid-Beta: Mechanistic Insights. Nutrients.

[65] Kashyap M.P., Roberts C., Waseem M., Tyagi P. (2018). Drug Targets in Neurotrophin Signaling in the Central and Peripheral Nervous System. Mol. Neurobiol..

[66] Bahdoudi S., Ghouili I., Hmiden M., do Rego J.L., Lefranc B., Leprince J., Chuquet J., do Rego J.C., Marcher A.B., Mandrup S. (2018). Neuroprotective effects of the gliopeptide ODN in an in vivo model of Parkinson’s disease. Cell. Mol. Life Sci..

[67] Schiborr C., Eckert G.P., Rimbach G., Frank J. (2010). A validated method for the quantification of curcumin in plasma and brain tissue by fast narrow-bore high-performance liquid chromatography with fluorescence detection. Anal. Bioanal. Chem..

[68] Ferri P., Angelino D., Gennari L., Benedetti S., Ambrogini P., Del Grande P., Ninfali P. (2015). Enhancement of flavonoid ability to cross the blood-brain barrier of rats by co-administration with α-tocopherol. Food Funct..

[69] Ulrich R.G. (2003). The toxicogenomics of nuclear receptor agonists. Curr. Opin. Chem. Biol..

[70] Schubert D., Humphreys S., Baroni C., Cohn M. (1969). In vitro differentiation of a mouse neuroblastoma. Proc. Natl. Acad. Sci. USA.

[71] Seeds N.W., Gilman A.G., Amano T., Nirenberg M.W. (1970). Regulation of axon formation by clonal lines of a neural tumor. Proc. Natl. Acad. Sci. USA.

[72] Evangelopoulos M.E., Weis J., Krüttgen A. (2005). Signalling pathways leading to neuroblastoma differentiation after serum withdrawal: HDL blocks neuroblastoma differentiation by inhibition of EGFR. Oncogene.

[73] Hamdi Y., Kaddour H., Vaudry D., Leprince J., Zarrouk A., Hammami M., Vaudry H., Tonon M.C., Amri M., Masmoudi-Kouki O. (2015). Octadecaneuropeptide ODN prevents hydrogen peroxide-induced oxidative damage of biomolecules in cultured rat astrocytes. Peptides.

[74] Ghouili I., Bahdoudi S., Morin F., Amri F., Hamdi Y., Coly P.M., Walet-Balieu M.L., Leprince J., Zekri S., Vaudry H. (2018). Endogenous Expression of ODN-Related Peptides in Astrocytes Contributes to Cell Protection against Oxidative Stress: Astrocyte-Neuron Crosstalk Relevance for Neuronal Survival. Mol. Neurobiol..

[75] Almeida A.S., Vieira H.L.A. (2017). Role of cell metabolism and mitochondrial function during adult neurogenesis. Neurochem. Res..

[76] Xie K., Sheppard A. (2018). Dietary micronutrients promote neuronal differentiation by modulating the mitochondrial-nuclear dialogue. Bioessays.

[77] Campbell G.R., Worrall J.T., Mahad D.J. (2014). The central role of mitochondria in axonal degeneration in multiple sclerosis. Mult. Scler. J..

[78] Sandoval-Acuña C., Ferreira J., Speisky H. (2014). Polyphenols and mitochondria: An update on their increasingly emerging ROS-scavenging independent actions. Arch. Biochem. Biophys..

[79] Sedel F., Bernard D., Mock D.M., Tourbah A. (2016). Targeting demyelination and virtual hypoxia with high-dose biotin as a treatment for progressive multiple sclerosis. Neuropharmacology.

[80] Notario B., Zamora M., Viñas O., Mampel T. (2003). All-trans-retinoic acid binds to and inhibits adenine nucleotide translocase and induces mitochondrial permeability transition. Mol. Pharmacol..

[81] Patil S.P., Jain P.D., Sancheti J.S., Ghumatkar P.J., Tambe R., Sathaye S. (2014). Neuroprotective and neurotrophic effects of Apigenin and Luteolin in MPTP induced parkinsonism in mice. Neuropharmacology.

[82] Qi G., Mi Y., Wang Y., Li R., Huang S., Li X., Liu X. (2017). Neuroprotective action of tea polyphenols on oxidative stress-induced apoptosis through the activation of the TrkB/CREB/BDNF pathway and Keap1/Nrf2 signaling pathway in SH-SY5Y cells and mice brain. Food Funct..

