# Supplementary material for: Induction of Neuronal Differentiation of Murine N2a Cells by Two Polyphenols Present in the Mediterranean Diet Mimicking Neurotrophins Activities: Resveratrol and Apigenin
Source: Diseases. 2018 Jul 22;6(3):67. doi: 10.3390/diseases6030067 (PMC6165409; doi:10.3390/diseases6030067)
Supplement: Supplementary file 1 [file diseases-06-00067-s001.pdf]

N2a: pre-cultured for 24h and treated for 48h with ODN in culture medium without or with 10% FBS

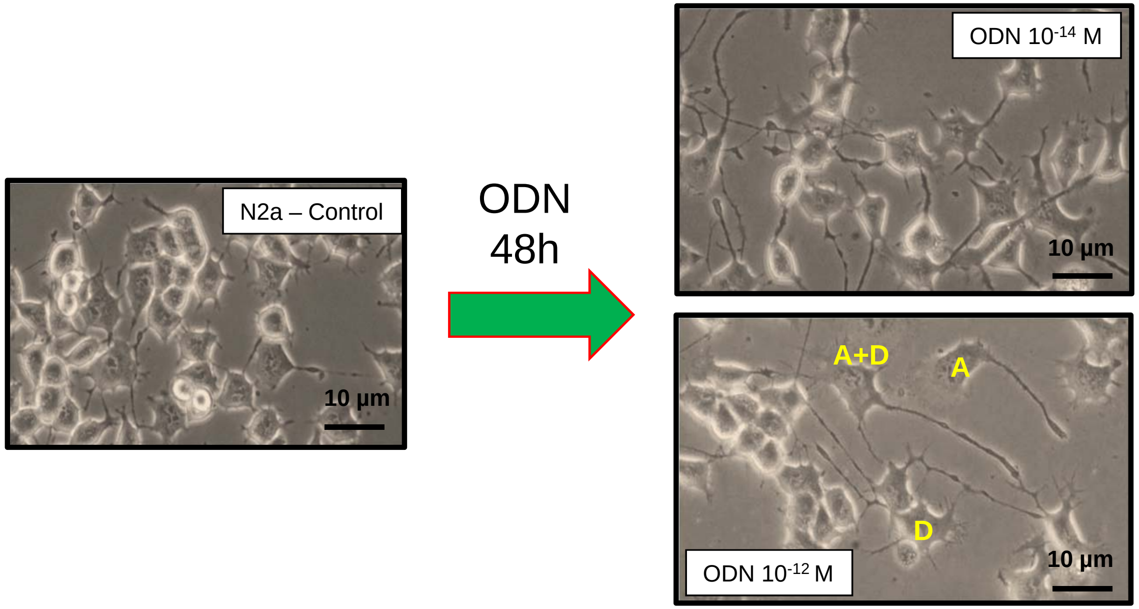

N2a expressing functionnal neurotrophins receptors (octadecaneuropeptide (ODN) receptors) have the ability to differentiate in neurons with dendrites (D), axons (A) and dendrites + axons (D + A)

Supplementary Figure 1, NAMSI et al.

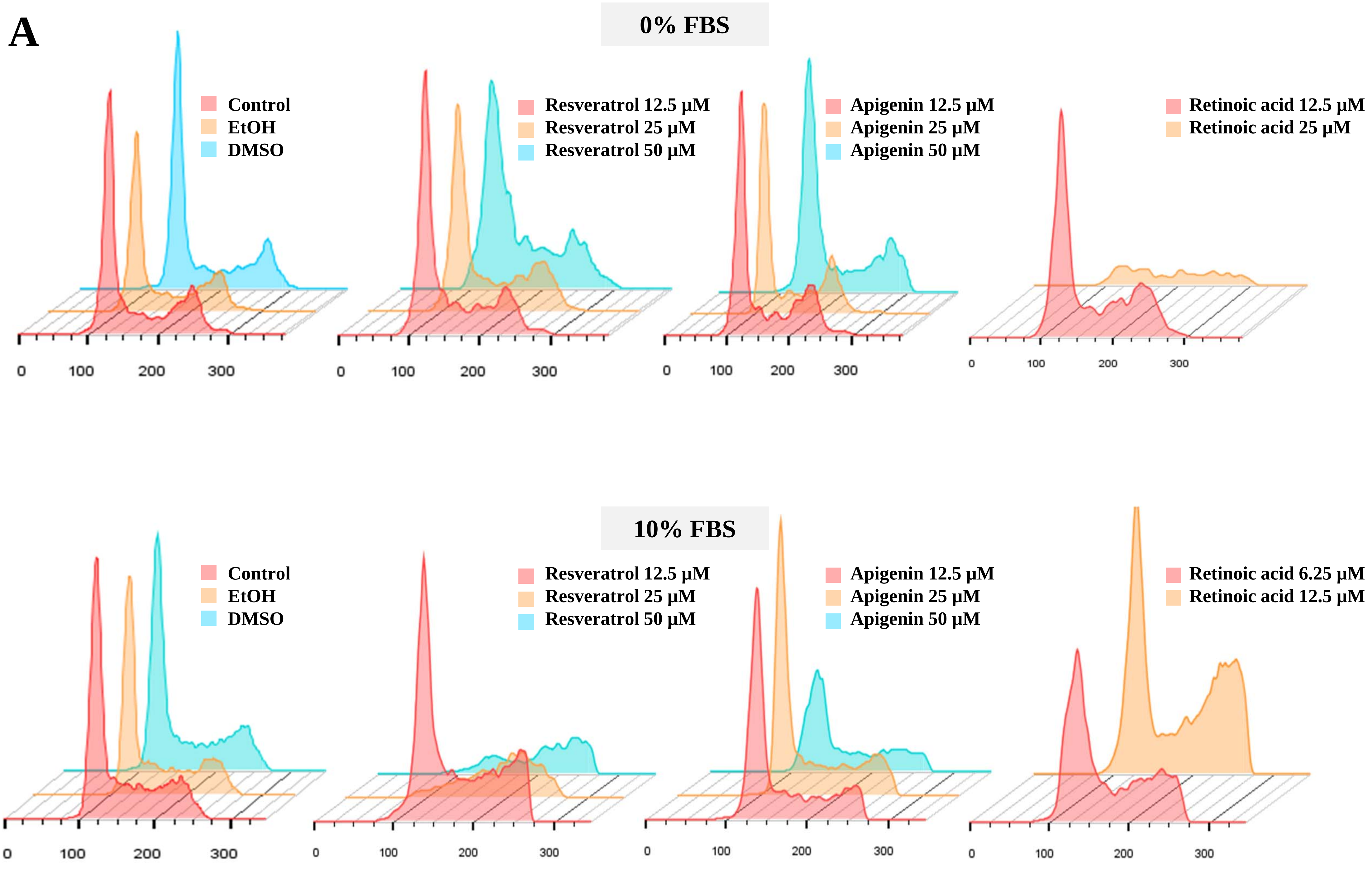

Supplementary Figure 2A, NAMSI et al

Repartition of the cells in the different phases of the cell cycle under different conditions of treatments with resveratrol (RSV), apigenin (API) and retinoic acid (RA) in culture medium without Fetal Bovine Serum (0% FBS) or with 10% FBS

FBS	Phases of the cell cycle	Treatments											
		Control	Vehicle (DMSO)	Vehicle (EtOH)	RSV 12.5 µM	RSV 25 µM	RSV 50 µM	API 12.5 µM	API 25 µM	API 50 µM	RA 6.25 µM	RA 12.5 µM	RA 25 µM
0%	G0/G1	*62*	*60*	*60*	*63*	*52*	*49*	*60*	*56*	*63*	*ND*	*47*	*25*
	S	*11*	*13*	*17*	*13*	*15*	*23*	*11*	*14*	*12*	*ND*	*28*	*27*
	G2 + M	*27*	*27*	*23*	*24*	*33*	*28*	*29*	*30*	*25*	*ND*	*35*	*48*
10%	G0/G1	*60*	*59*	*58*	*46*	*7*	*10*	*55*	*50*	*51*	*48*	*34*	*ND*
	S	*15*	*19*	*20*	*22*	*65*	*73*	*20*	*27*	*35*	*45*	*14*	*ND*
	G2 + M	*25*	*22*	*22*	*32*	*28*	*17*	*25*	*23*	*14*	*17*	*52*	*ND*

ND: not determined

Supplementary Figure 2B, NAMSI et al

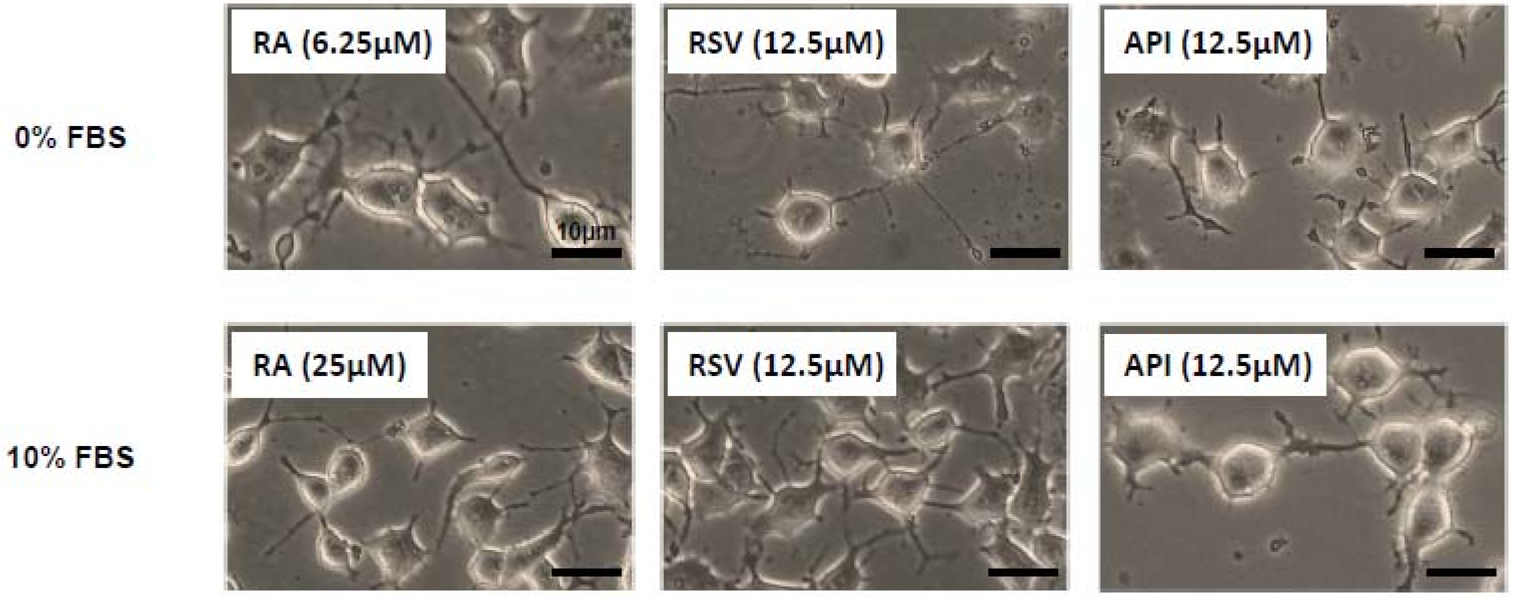

Supplementary Figure 3, NAMSI et al
